# Long Non-Coding RNAs in *Cryptococcus neoformans*: Insights Into Fungal Pathogenesis

**DOI:** 10.3389/fcimb.2022.858317

**Published:** 2022-03-17

**Authors:** Murat C. Kalem, John C. Panepinto

**Affiliations:** Department of Microbiology and Immunology, Witebsky Center for Microbial Pathogenesis and Immunology, Jacobs School of Medicine and Biomedical Sciences, State University of New York (SUNY), University at Buffalo, Buffalo, NY, United States

**Keywords:** lncRNA, cryptococcus, fungi, pathogenesis, genome

## Abstract

Long non-coding RNAs (lncRNAs) are highly expressed and can modulate multiple cellular processes including transcription, splicing, translation, and many diverse signaling events. LncRNAs can act as sponges for miRNAs, RNA and DNA binding proteins, functioning as competitive endogenous RNAs. The contribution of lncRNAs to microbial pathogenesis is largely neglected in eukaryotic pathogens despite the abundance of RNA sequencing datasets encompassing conditions of stress, gene deletions and conditions that mimic the host environment. The human fungal pathogen *Cryptococcus neoformans* encodes 6975 (84%) protein-coding and 1359 (16%) non-protein-coding RNAs, of which 1182 (14.2%) are lncRNAs defined by a threshold of greater than 200 nucleotides in length. Here, we discuss the current state of knowledge in *C. neoformans* lncRNA biology. Utilizing existing RNA seq datasets, we examine trends in lncRNA expression and discuss potential implications for pathogenesis.

## Introduction

Long non-coding RNAs (lncRNAs) were first thought to be transcriptional noise or junk RNAs. Although they are named for what they do not do – encode for proteins – we now know that lncRNAs have an impressive range of biological functions (Ponting et al., 2009; Rinn and Chang, 2012; Scaria and Pasha, 2013; Akhade et al., 2017; Zhang et al., 2019). The central dogma of molecular biology states that genetic information is transferred from DNA to RNA to protein, creating an incomplete narrative that an RNA molecule only has value if it encodes for a protein. This dogma, accepted as the backbone of molecular biology, constrains our vision of the vast functional diversity that exists among RNA molecules. Over the last two decades, this dogma has been challenged by non-coding RNAs and major advances in the field of RNA biology.

Non-coding RNAs include a diverse cohort of RNA molecules that can be categorized under two broad groups: housekeeping ncRNAs and regulatory ncRNAs. Housekeeping ncRNAs include ribosomal RNAs (rRNAs), transfer RNAs (tRNAs), small nuclear RNAs (snRNAs), small nucleolar RNAs (snoRNAs), and tRNA fragments (tRFs). Regulatory ncRNAs include micro RNAs (miRNAs), small interfering RNAs (siRNAs), circular RNAs (circRNAs), and long non-coding RNAs (lncRNAs) (Rinn and Chang, 2012; Akhade et al., 2017; Zhang et al., 2019). LncRNAs are classified as non-coding RNA molecules that are larger than 200 bases. Classification of the lncRNAs solely based on length is arbitrary. To broaden this definition, it is proposed that non-coding RNAs that function as primary or spliced RNAs outside of the known small ncRNA classes are also considered lncRNAs (Amaral et al., 2011). Consequently, there are some lncRNAs that do not meet the arbitrary length threshold such as *BC1* and *snaR* (Parrott and Mathews, 2007; Kim et al., 2016).

Steps in RNA metabolism from mRNA transcription to maturation might be differentially modulated for lncRNAs. As products of RNA polymerase II transcription, lncRNAs may or may not be processed through canonical spliceosome-dependent splicing, 3’ end processing and polyadenylation. For example, human lncRNAs MALAT1 and NEAT1_2 have 3’ triple helix structures instead of poly-A tails (Brown et al., 2012; Wilusz et al., 2012). This suggests that alternatively processed lncRNAs may have unique post-transcriptional regulatory mechanisms, possibly through RNA-binding proteins that recognize and bind to these unique structural elements. Interestingly, MALAT1 3’ triple helix structures are shown to engage with ribosomes suggesting a potential role in translation regulation (Bhandari et al., 2014). This type of poly-A-independent mechanism is not uncommon, and in fact, histone mRNAs contain 3’ stem-loops instead of poly-A tails and are regulated by the binding of stem-loop binding proteins (Wang et al., 1996).

LncRNAs localize to both the cytoplasm and the nucleus, therefore they are able to regulate molecular processes across multiple cellular locations (Cabili et al., 2015; Dunagin et al., 2015; Miao et al., 2019). They can regulate gene expression at the level of transcription, can impact post-transcriptional processes, and modulate translation. LncRNAs can act as a guide for chromatin-modifying enzymes, and as decoys for RNA or DNA binding proteins or other signaling molecules (Wang and Chang, 2011; Chacko and Lin, 2013; Akhade et al., 2017). They can act as endogenous competitors, or as sponges for miRNAs, and they themselves can be precursors that are processed to form small RNAs (Wang and Chang, 2011; Sweta et al., 2019). Many lncRNAs lack a canonical open reading frame, yet there is recent evidence that some lncRNAs produce micropeptides with functional roles (Hartford and Lal, 2020). For example, micropeptide Myoregulin modulates muscle development in mice and another micropeptide Sda controls sporulation in *Bacillus subtilis* (Burkholder et al., 2001; Anderson et al., 2016).

A single lncRNA can exert robust and widespread molecular control in cellular processes; one great example is the lncRNA Xist which can silence an entire chromosome by recruiting necessary effector proteins (Sunwoo et al., 2015; Jachowicz et al., 2021). Studies in mammalian cells and model systems implicate lncRNAs as important players in cellular and molecular processes and demonstrate their impact on disease pathologies including cancers, neurodegenerative and autoimmune diseases (Gao et al., 2018; Wang et al., 2018; Piccolo et al., 2019; Zhou et al., 2021). Despite their importance, the contribution of lncRNAs to eukaryotic gene regulation in fungi, especially in fungal pathogens, is a neglected area of study.

Here, we aim to briefly highlight some findings on the importance of lncRNAs in pathogenic fungi and then focus on lncRNAs in the human fungal pathogen *Cryptococcus neoformans*. In this perspective, we curate the lncRNAs of *C. neoformans* and utilize the published RNA sequencing data sets to investigate lncRNA expression under conditions that mimic host microenvironments. Given their large numbers and dynamic expression across stress conditions, we are hopeful that further investigation into lncRNA function will identify lncRNA molecules involved in fungal-specific virulence factor production, stress adaptation, and pathogenesis.

## Non-Coding RNAs in Fungal Pathogens

Although studies of lncRNAs in fungi are not as abundant as in other kingdoms of life, there is evidence that lncRNAs have regulatory roles in many fungal species (Chacko and Lin, 2013; Till et al., 2018; Dhingra, 2020; Li et al., 2021). A majority of the knowledge on lncRNAs in fungi comes from the model yeasts *S. cerevisiae* and *S. pombe* (Yamashita et al., 2016). In *Saccharomyces cerevisiae*, there are both cis-acting lncRNAs, which regulate neighboring genes at a single genetic locus, and trans-acting lncRNAs, which can exert regulatory function outside of the genetic locus that encodes it. For example, *CDC28*-antisense lncRNA, *pHO*-lncRNA, *PWR1*, *ICR1*, *RME2*, and *RME3* are identified to be cis-acting since ectopic expression of these lncRNAs presents a loss-of-function due to the absence of lncRNA-mediated cis-regulation (Hongay et al., 2006; Bumgarner et al., 2009; Nadal-Ribelles et al., 2014; Yu et al., 2016; Till et al., 2018). Cis-acting *RME2* and *RME3* have unique functions in regulating meiosis (Hongay et al., 2006). Although trans-acting lncRNAs are less common in *S. cerevisiae* than cis-acting lncRNAs, *SUT457* is an example of an *S. cerevisiae* trans-acting lncRNA that interacts with 12 target genes that are important for telomere organization (Kyriakou et al., 2016).

Recent studies also identified lncRNAs in the plant pathogens *Fusarium graminearum* and *Magnaporthe oryzae*, the insect pathogen ﻿*Metarhizium robertsii*, the model organism *Neurospora crassa*, and human fungal pathogens *Candida auris*, *Aspergillus fumigatus*, and *Cryptococcus neoformans*. In the plant pathogenic fungus *Fusarium graminearum*, lncRNAs exhibit dynamic expression patterns during sexual fruiting body formation, suggesting a regulatory role for lncRNAs in fungal development (Kim et al., 2018). In the insect pathogen *Metarhizium robertsii*, lncRNAs are regulated during heat shock, 1085 out of 1655 lncRNAs were shown to be differentially expressed, both up- and down-regulated, following heat stress (Wang et al., 2019). One specific lncRNA, *mrLncRNA1*, plays a role in conidial thermotolerance, and in the absence of *mrLncRNA1, M. robertsii* exhibits a defect in germination (Wang et al., 2019). Bioinformatic analysis of lncRNAs in *Candida* pathogens (*C. albicans*, *C. tropicalis*, *C. parapsilosis*, *C. auris*, and *C. glabrata*) demonstrated the co-regulation of lncRNA expression with certain protein-coding genes while identifying many differentially regulated lncRNAs during the infection of human epithelial cells (Hovhannisyan and Gabaldón, 2021). Another important fungal lncRNA was identified during a piggyBac transposon mutagenesis screen in *C. auris* that resulted in a mutant that exhibited constitutive filamentous growth (Gao et al., 2021). This mutant had a transposon insertion in a lncRNA gene named DNA damage inducible lncRNA – DINOR (Gao et al., 2021). Deletion of DINOR resulted in DNA damage exhibited as fragmented DNA and caused hyperphosphorylation of kinase Rad53 while leading to attenuated virulence in mice. DINOR expression was upregulated in the presence of DNA damaging alkylating agents, antifungals such as amphotericin B and fluconazole, and peroxide-induced oxidative stress (Gao et al., 2021). The discovery of DINOR, along with other studies highlighting the importance of lncRNAs in fungal pathogens, provides a compelling proof-of-concept that lncRNAs can have robust roles in fungal virulence.

In *Cryptococcus neoformans*, there is only one lncRNA with an identified function. LncRNA *RZE1* was identified through a forward genetic screen in *C. neoformans* to identify upstream regulators of Znf2 (Chacko et al., 2015). *ZNF2* deletion locks *C. neoformans* in the yeast form causing increased virulence. Conversely, *ZNF2* overexpression results in hyperfilamentous phenotype and decreased virulence. LncRNA *RZE1* exhibits mainly nuclear localization and exerts its function on the *ZNF2* mRNA. *RZE1* deletion results in decreased *ZNF2* transcript levels and altered subcellular localization of the *ZNF2* mRNA (Chacko et al., 2015). It is likely that more than one lncRNA in *C. neoformans* exerts biological function, and the remaining unstudied lncRNAs may represent unique fungal biology, and importantly, novel virulence regulatory mechanisms. In the rest of this perspective, we present the characterization of *C. neoformans* lncRNAs and explore their expression profiles in published RNA sequencing studies. We believe that studying lncRNA biology will provide novel insights into *Cryptococcal* pathogenesis and regulation of virulence.

## Characterization of Long Non-Coding RNAs in *Cryptococcus neoformans*

**Figure 1:**
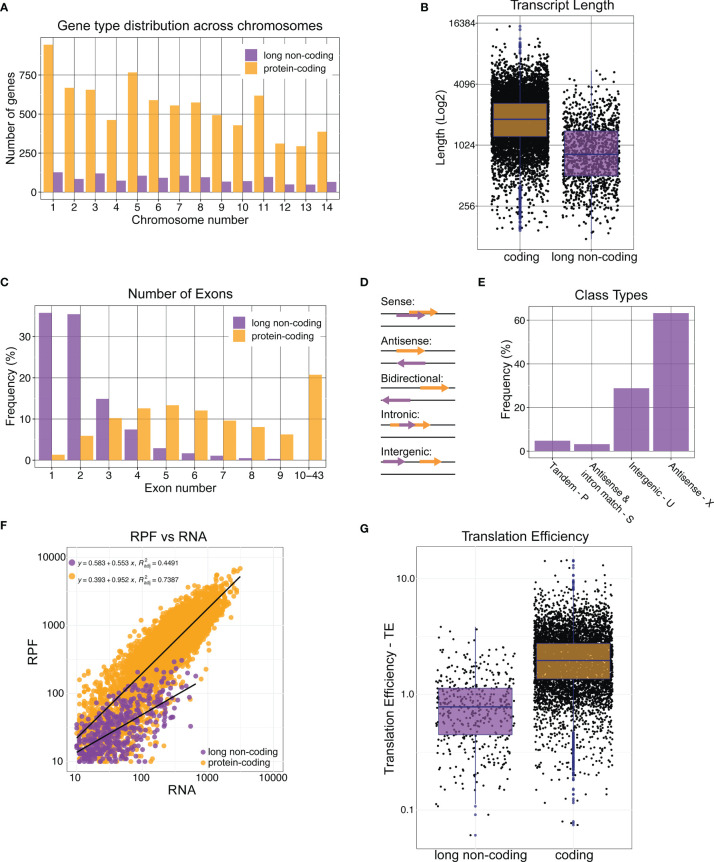
Characterization of annotated long non-coding RNAs in *Cryptococcus neoformans*. *C. neoformans* (strain H99) non-coding RNA sequences were obtained from FungiDB. We investigated the genomic locations of lncRNAs across chromosomes **(A)**, transcript length **(B)**, and the number of exons **(C)**. LncRNAs are classified based on their juxtaposition relative to protein-coding genes. We determined the lncRNA class types using the GffCompare program **(D, E)**. We investigated the occupancy of lncRNAs by ribosomes by plotting ribosome-protected fragments (RPF) vs. RNA abundance using a 10 reads minimum threshold. Regression analysis, shown as black lines, and corresponding equations with R^2^ values are also presented **(F)**. Translation efficiency was calculated by using the RPF/RNA formula and plotted for both coding and non-coding RNAs **(G)**. Ribosome profiling data is obtained from GSE133125. Bed files supplied by the authors were utilized to obtain the coding and non-coding transcript counts.

To begin with, we analyzed the *Cryptococcus neoformans* genome for annotated lncRNAs using the annotations on FungiDB (Basenko et al., 2018). *C. neoformans* encodes for 6975 (84%) protein-coding and 1359 (16%) non-protein-coding RNAs, of which 1182 (14.2%) are lncRNAs with a 200-nucleotide length threshold. We scrutinized the various features of coding and non-coding transcripts. First, we looked at the distribution of the lncRNA encoding genes across the genome and found that they are equally distributed across 14 chromosomes with no apparent enrichment (**Figure 1A**). The average transcript size for coding and long non-coding RNAs revealed that lncRNAs are on average shorter than mRNAs (**Figure 1B**). Despite not being translated, lncRNAs can still undergo splicing and other RNA processing steps. We analyzed the number of exons in coding and non-coding transcripts. This analysis revealed that more than ~60% percent of non-coding RNAs either contain a single exon (no intron) or very few exons in comparison to mRNAs (**Figure 1C**). RNA sequencing evidence suggests that lncRNA introns are as efficiently spliced as most mRNAs. LncRNAs do not have an accumulation of intronic sequences. LncRNAs can be classified based on their relative location to protein-coding genes (**Figure 1D**). These classes are 1) sense – lncRNA overlaps another gene on the same strand, 2) antisense – lncRNA overlaps another gene on the opposite strand, 3) bidirectional – transcription of lncRNA and another gene on the opposite strand are initiated in close proximity, 4) intronic – full lncRNA is derived from an intron, 5) intergenic – lncRNA exists between multiple genes. Investigations of these different class types using GffCompare and annotations on FungiDB resulted in that majority of the lncRNAs in *C. neoformans* are antisense lncRNAs that have exonic overlap with the genes on the opposite strand (Pertea and Pertea, 2020). This was followed by the intergenic lncRNAs being the second most common class type in *C. neoformans* lncRNAs (**Figure 1E** and **Supplementary File 1**). This suggests a profound possibility that lncRNA expression in *C. neoformans* can possess regulation in *cis* and impact the expression of neighboring and overlapping genes.

Following this initial characterization of *C. neoformans* lncRNAs, we utilized the previously published ribosome profiling and RNA sequencing data sets to ask if any of the identified lncRNAs are ribosome-associated (Wallace et al., 2020). Of note, our analysis also includes some shorter lncRNAs as lncRNA length classification is arbitrary. Analysis of the ribosome protected fragments (RPFs) and RNA abundance revealed that while protein-coding transcripts have a more linear correlation between RPFs and RNA abundance ( ${\text{R}}_{\text{adj}}^{2}$ : 0.7378), long non-coding transcripts do not have as robust of a relationship between RPFs and RNA abundance ( ${\text{R}}_{\text{adj}}^{2}$ : 0.4491) (**Figure 1F** and **Supplementary File 1**). Following this, we calculated the translation efficiency (TE) by dividing the RPF read count by RNA read count. This analysis revealed that although long non-coding RNAs have lower average TE values, there are some ncRNAs with TE values as high as that of protein-coding transcripts (**Figure 1G** and **Supplementary File 1**). One can speculate multiple scenarios that could explain this. First, these lncRNAs might be misannotated and they might encode unstable peptides that are yet to be identified. Second, ribosome profiling experiments include ribosome purification and nuclease digest steps to selectively isolate the RNA fragments protected by ribosomes. Despite all efforts to only isolate ribosome-bound RNAs, there might be some RNAs that are protected from the nuclease digest by a large protein complex such as RNA-binding protein complexes. And third, these ncRNAs with high TE values might actually be bound by ribosomes and act as endogenous competitive RNAs to occupy and eliminate ribosomes from translating other transcripts. These hypotheses provide exciting possibilities for the identification of fungal-specific gene regulatory mechanisms as most lncRNAs in *Cryptococcus* do not seem to be conserved across kingdoms based on BLAST analysis.

## Harnessing the Power of Next-Generation Sequencing to Investigate lncRNA Expression in *Cryptococcus neoformans*

RNA sequencing became one of the most popular and commonly utilized methods over the past two decades. This widely-appreciated technological advance not only allowed scientists to appreciate the transcriptome-wide gene expression patterns but also created this enormous pool of data that can be mined to address many neglected hypotheses - one of which is that fungal lncRNAs have dynamic expression patterns and are important in modulating virulence and virulence-associated traits in the fungal pathogen *C. neoformans*. We investigated the expression of lncRNAs in *C. neoformans* in response to infection-relevant stressors using published data sets. Some of the major stressors and unique microenvironments that environmental fungi encounter during infection include elevated temperature, growth within macrophages in the presence of oxidative stress insult, and growth within the central nervous system such as cerebrospinal fluid (CSF). For that reason, we utilized RNA sequencing data obtained from *C. neoformans* cells phagocytosed by activated J774A murine macrophages following 2 hours co-infection, *C. neoformans* isolated from corticosteroid treated New Zealand white rabbit CSF following 24 hours post intracisternal inoculation, and *C. neoformans* cells grown at 30°C and shifted to 37°C for one hour (Bloom et al., 2019; Yu et al., 2020).

**Figure 2:**
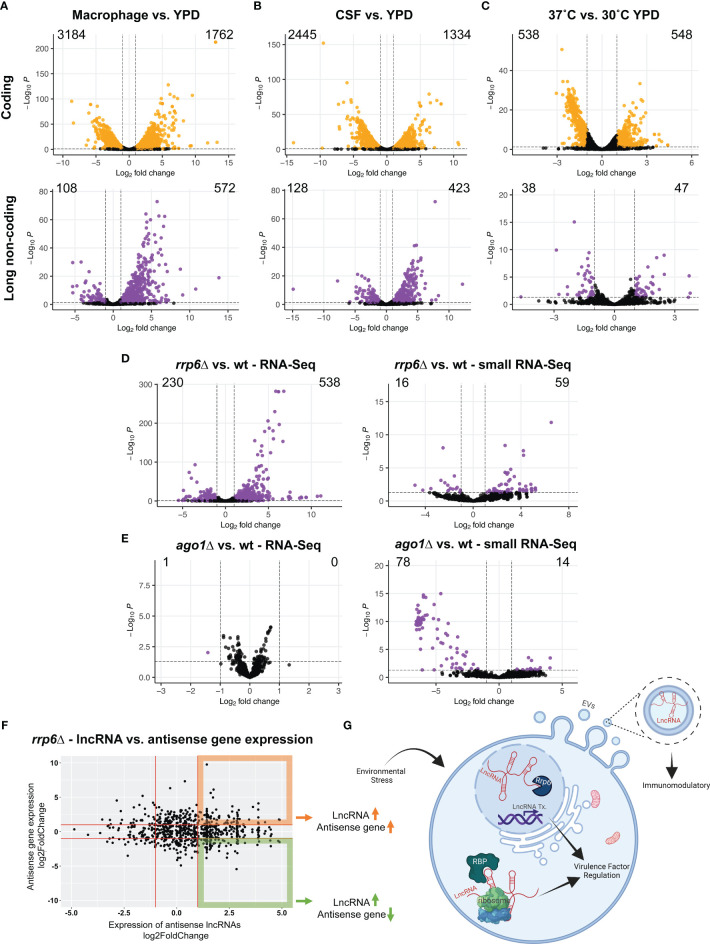
Long non-coding RNA expression under conditions that mimic the host environment and in gene deletion mutants. We utilized the public datasets to investigate the expression of lncRNAs under conditions that are relevant to infection. These conditions include *in vitro* J774A macrophage infection **(A)**, CSF **(B)**, and 37˚C compared to 30˚C in YPD **(C)**. The number of upregulated and downregulated genes were noted on each graph for both coding (orange) and non-coding (purple) genes. We then show the impact of *RRP6* **(D)** and *AGO1* **(E)** gene deletions on lncRNA expression as well as changes in the expression of small RNAs that map to full-length lncRNAs. We show the expression of antisense lncRNAs and antisense protein-coding transcripts in the *rrp6*Δ **(F)**. RNA sequencing data were obtained from GSE136879, GSE121183 and GSE128009. GSE136879 data files were trimmed and mapped to the H99 genome using STAR alignment and gene expression analysis was performed using RSEM and DeSeq2. GSE121183 and GSE128009 data were analyzed for ncRNA expression using the supplementary tables provided by the authors of these studies. **(G)** Graphical summary/model. LncRNAs can have unique functions in distinct cellular and extracellular compartments that impact pathogenesis and immune modulation. The graphical summary is created using biorender.com.

Analysis of the expression of both coding and long non-coding RNAs revealed massive remodeling of both kinds of transcripts. The macrophage condition had 572 upregulated and 108 downregulated lncRNAs compared to YPD (**Figure 2A** and **Supplementary File 1**). The CSF condition had 423 upregulated and 128 downregulated lncRNAs compared to YPD (**Figure 2B** and **Supplementary File 1**). Under elevated temperature challenge, we identified 47 upregulated and 38 downregulated lncRNAs with a 0.05 adjusted p-value and 2-fold change threshold (**Figure 2C** and **Supplementary File 1**). These results suggest that lncRNA expression, along with mRNA expression, is dynamic under infection-relevant stress conditions and likely crucial in fine-tuning gene regulatory or signaling pathways necessary to adapt to constantly and rapidly changing environments.

In addition to RNA sequencing data sets from host-relevant conditions, there is an abundance of transcriptome-wide analyses from many gene deletion mutants of *C. neoformans*. Investigations of lncRNA expression in these mutants can yield the determination of the key factors that control lncRNA expression. Expression of lncRNAs in gene deletion mutants along with the phylogenetic conservation of deleted gene products could hint us at the evolutionarily conserved or fungi-specific mechanisms of lncRNA expression modulation.

We utilized published *rrp6*Δ and *ago1*Δ RNA sequencing data sets and determined the expression of lncRNAs (Burke et al., 2019). Rrp6 is a 3’ to 5’ exoribonuclease and a critical component of the nuclear RNA exosome which is the complex responsible for catalyzing degradation, processing, surveillance, and export of coding and non-coding RNAs (Davis and Ares, 2006; Zhang et al., 2014; Fox and Mosley, 2016). The RNA exosome complex regulates the maturation of some housekeeping ncRNAs such as rRNAs, snoRNAs, and snRNAs (Fox and Mosley, 2016). It has been shown that mutations in Rrp6 yield aberrant polyadenylation of snoRNA transcripts in yeast and these misregulated transcripts are then rapidly degraded in an Rrp6-dependent fashion (Grzechnik and Kufel, 2008). Substrates of Rrp6 include many non-coding RNAs (Chacko et al., 2015). In humans, Rrp6 deletion results in increased transcription upstream of promoters, called promoter upstream transcripts or PROMPTs. PROMPTs are stabilized in exosome depleted cells and some even have functions (Preker et al., 2008). Rrp6-mediated RNA decay has shown to be important for eliminating many non-coding RNAs that arise from pervasive transcription and transcriptional noise (Davis and Ares, 2006). Another study highlights that an Rrp6-like protein in *Arabidopsis* maintains DNA methylation through retention of lncRNAs, which provides an example in which Rrp6 can positively regulate lncRNA expression (Zhang et al., 2014). Analysis of lncRNA expression in the *C. neoformans rrp6*Δ mutant revealed that 538 lncRNAs were upregulated and 230 were downregulated in the mutant compared to wildtype (**Figure 2D**). This suggests that while the majority of lncRNAs are upregulated in the absence of Rrp6, the nuclear exosome might modulate lncRNA expression both negatively and positively.

Another dataset we investigated was from the Argonaute 1 (Ago1) deletion mutant (Burke et al., 2019). Ago1 binds to short RNAs and is a critical component of the RNAi pathway and small RNA mediated gene silencing (Williams and Rubin, 2002). LncRNAs can act as precursors for miRNAs and small RNAs (Arikit et al., 2013). Since the majority of the lncRNAs are antisense to other coding genes, we reasoned that small RNAs generated from lncRNAs could regulate the expression of coding genes expressed on the opposite strand through small RNA mediated gene silencing. We analyzed the expression of ncRNAs and small RNAs that map to full-length ncRNAs in the *ago1*Δ mutant. Interestingly, this mutant did not impact the expression of lncRNAs, however, the mutant had 14 upregulated and 78 downregulated small RNAs that map to lncRNAs (**Figure 2E**). This suggests that Ago1 might bind to small RNAs that are generated from lncRNAs, and in the absence of Ago1 these small RNAs are likely degraded or dysregulated. Investigation of small RNAs originating from lncRNAs in the *rrp6*Δ showed that 59 were upregulated and 16 were downregulated (**Figure 2E**).

Since many *C. neoformans* lncRNAs are antisense to protein-coding genes, we investigated the expression of lncRNAs and their corresponding antisense protein-coding genes in the *rrp6*Δ (**Figure 2F**). Our analysis revealed that upregulated antisense lncRNAs can both positively and negatively influence the expression of the antisense protein-coding gene. This suggests that Rrp6-mediated lncRNA expression regulation may alter the antisense gene expression in a context-dependent manner. These observations utilizing published RNA sequencing datasets will allow us to generate testable hypotheses to explore lncRNA biology in *C. neoformans*.

## Discussion

Because evolution is an opportunistic process, we can speculate that a majority of lncRNAs likely have functions otherwise they would not exist. Even though lncRNAs are not highly conserved in terms of linear sequence homology, conserved biological functions, and even structures, are likely under evolutionary selection (Johnsson et al., 2014; Ulitsky, 2016; Ramírez-Colmenero and Fernandez-Valverde, 2020). LncRNAs *DINOR* and *RZE1* are the best examples highlighting the functional roles of lncRNAs in fungi yet they only represent the tip of the iceberg (Chacko et al., 2015; Gao et al., 2021). Both *RZE1* and *DINOR* are only conserved within their respective species complexes based on sequence analyses, yet this does not exclude the possibility of functional conservation without retaining sequence homology.

Advances in sequencing technologies revealed the diversity of RNA molecules and allowed us to investigate the expression of coding and non-coding transcripts under various conditions. However, post-transcriptional processing of lncRNAs is unclear, and RNA-sequencing experiments including a poly-A purification method may exclude a subset of lncRNAs that are either non-polyadenylated or are conditionally polyadenylated. With this in mind, all of the data sets utilized in our analyses are biased for polyadenylated RNAs. Optimization and widespread utilization of efficient RNase H based rRNA depletion methods utilizing antisense oligonucleotides will improve our understanding of the diverse elements within fungal transcriptomes including non-coding RNAs. Recent developments in the field revealed that RNase H based rRNA removal is efficient in C*. neoformans* and analysis of lncRNAs using LncPipe following an RNase H based rRNA depletion method revealed 11 novel lncRNAs (Telzrow et al., 2021). Our brief analysis of lncRNA expression utilizing published sequencing data sets is not able to determine the difference between changes in expression versus polyadenylation or deadenylation. Future work investigating the non-coding RNA processing networks under host-relevant stress conditions could reveal unique fungal biology, including pathways for lncRNA maturation and processing.

*C. neoformans* secretes extracellular vesicles containing components of the polysaccharide capsule - one of its virulence factors - that are important for macrophage modulation (Rodrigues et al., 2008; Oliveira et al., 2010). Additionally, *C. neoformans* EVs are packaged with protein and RNA cargo (Da Silva et al., 2015; Rizzo et al., 2021). The RNA cargo includes a wide variety of RNA molecules including extracellular RNAs (exRNAs), miRNAs, siRNAs and lncRNAs (Liu et al., 2020). We can speculate that the secreted RNA cargo might have an immunomodulatory role during infection or amoeba predation (**Figure 2G**). For example, a deployed small silencing RNA from entomopathogenic fungus *Beauveria bassiana* binds to host cell Ago1 and hijacks the host RNA interference machinery (Cui et al., 2019). Further studies are needed to explore the immunomodulatory mechanisms of secreted non-coding RNAs of *C. neoformans.*

This brief characterization and expression analysis highlight the dynamic expression patterns of lncRNAs in *C. neoformans*. We hypothesize that changes in lncRNA expression may result in altered nuclear and cytoplasmic post-transcriptional events that impact virulence factors (**Figure 2G**). We also suggest that lncRNAs play functional roles in regulating *C. neoformans* pathogenesis (**Figure 2G**). Future studies focusing on individual lncRNAs will delineate functions for each lncRNA in modulating cellular processes important for virulence and pathogenesis. Even though the majority of the *C. neoformans* lncRNAs are antisense to a protein-coding gene which makes it impossible to generate specific deletions using homologous recombination, the latest advances in gene manipulations utilizing CRISPR Cas9 and Cas13 will make it possible to more efficiently knockout and knockdown lncRNAs, respectively. These tools could be utilized to create lncRNA knockout and knockdown libraries in order to screen for lncRNA involvement in virulence and pathogenesis as well as to identify lncRNA mutant strains that are sensitive or resistant to current antifungals. We are confident that the studies of lncRNAs in fungi will continue to be fruitful and lead to discoveries of unique fungal biology.

## Data Availability Statement

The original contributions presented in the study are included in the article/**Supplementary Material**. Further inquiries can be directed to the corresponding authors.

## Author Contributions

MCK conceptualized the piece, performed the analyses and drafted the manuscript. JCP edited the manuscript. Both authors contributed to the article and approved the submitted version.

## Funding

National Institute of Health (NIH) R01AI131977 to JCP. American Heart Association Fellowship Award ID 827110 to MCK.

## Conflict of Interest

The authors declare that the research was conducted in the absence of any commercial or financial relationships that could be construed as a potential conflict of interest.

## Publisher’s Note

All claims expressed in this article are solely those of the authors and do not necessarily represent those of their affiliated organizations, or those of the publisher, the editors and the reviewers. Any product that may be evaluated in this article, or claim that may be made by its manufacturer, is not guaranteed or endorsed by the publisher.
